# Sentinel Lymph Node Detection Using Carbon Nanoparticles in Patients with Early Breast Cancer

**DOI:** 10.1371/journal.pone.0135714

**Published:** 2015-08-21

**Authors:** Xiufeng Wu, Qingzhong Lin, Gang Chen, Jianping Lu, Yi Zeng, Xia Chen, Jun Yan

**Affiliations:** 1 Department of Surgery, Fujian Provincial Tumor Hospital, Teaching Hospital of Fujian Medical University, Fuzhou 350014, Fujian, People’s Republic of China; 2 Department of General Surgery, Nanfang Hospital, Southern Medical University, Guangzhou 510515, Guangdong, People’s Republic of China; 3 Department of Pathology, Fujian Provincial Tumor Hospital, Teaching Hospital of Fujian Medical University, Fuzhou 350014, Fujian, People’s Republic of China; The First Affiliated Hospital with Nanjing Medical University, CHINA

## Abstract

**Purpose:**

Carbon nanoparticles have a strong affinity for the lymphatic system. The purpose of this study was to evaluate the feasibility of sentinel lymph node biopsy using carbon nanoparticles in early breast cancer and to optimize the application procedure.

**Methods:**

Firstly, we performed a pilot study to demonstrate the optimized condition using carbon nanoparticles for sentinel lymph nodes (SLNs) detection by investigating 36 clinically node negative breast cancer patients. In subsequent prospective study, 83 patients with clinically node negative breast cancer were included to evaluate SLNs using carbon nanoparticles. Another 83 SLNs were detected by using blue dye. SLNs detection parameters were compared between the methods. All patients irrespective of the SLNs status underwent axillary lymph node dissection for verification of axillary node status after the SLN biopsy.

**Results:**

In pilot study, a 1 ml carbon nanoparticles suspension used 10–15min before surgery was associated with the best detection rate. In subsequent prospective study, with carbon nanoparticles, the identification rate, accuracy, false negative rate was 100%, 96.4%, 11.1%, respectively. The identification rate and accuracy were 88% and 95.5% with 15.8% of false negative rate using blue dye technique. The use of carbon nanoparticles suspension showed significantly superior results in identification rate (p = 0.001) and reduced false-negative results compared with blue dye technique.

**Conclusion:**

Our study demonstrated feasibility and accuracy of using carbon nanoparticles for SLNs mapping in breast cancer patients. Carbon nanoparticles are useful in SLNs detection in institutions without access to radioisotope.

## Introduction

Sentinel lymph node biopsy (SLNB) is regarded as standard of care for axillary nodal staging in clinically axillary node-negative breast cancer patients [1]. SLNB will ensure the need for axillary lymph node dissection (ALND) in patients with positive sentinel lymph nodes (SLNs) and is equivalent to ALND in terms of correct staging but is associated with less-extensive morbidity than ALND [2–4]. Currently, SLNB is dependent on injection of blue dye, radioactive colloid, the combination of both or indocyanine green (ICG). The identification rates vary with blue dye (68–86%), radioisotope (86–99%), combined technique (89–97%), ICG (73.8–99%) [5–11]. Despite high rates of sentinel lymph node (SLN) detection with these techniques, there is no general consensus about the optimal technique [12]. Radioactive colloid method results in some concerns about limited availability and cost of radio colloids, and radiation exposure [13]. In addition, lymphoscintigraphy with a radioactive colloid cannot provide real-time visual during the surgical procedure. Both blue dye and ICG, due to its relatively small diameter, permit flow through the SLNs to higher tier nodes, which results in incorrectly identifying sentinel nodes [14, 15]. Moreover, the ICG technique requires special equipment in the operating room to enable this procedure [16].

Carbon nanoparticles are a synthetic tracer via the specific modification of small activated carbon particles with an average diameter of 150 nm, which is widely used in the field of cancer diagnosis and therapy [17]. They have received considerable interest in recent years, especially with respect to their potential utilization of lymphatic mapping. Carbon nanoparticles selectively enter the lymphatic vessels rather than blood capillaries due to the molecular size and permeability. Upon injection into the tissues around the tumor, carbon nanoparticles are rapidly engulfed by macrophages and then pass through the lymphatic vessels to the SLNs, thus staining them black. This technique facilitates the vital staining of tumor-draining lymph nodes, and has been applied in the detection of sentinel lymph nodes (SLNs) in colorectal and thyroid cancers [18, 19]. Carbon nanoparticles have no toxic side effects on the human body due to less access to the blood circulation. Because of safety and strong affinity for the lymphatic system, carbon nanoparticles were approved for SLN mapping in gastric cancer by Chinese Food and Drug Administration. Therefore the feasibility of carbon nanoparticles for the identification of SLNs in early breast cancer must be investigated. In our study, the assessment of SLN detection using carbon nanoparticles is performed. We compare the identification rate, accuracy, false negative rate using carbon nanoparticles with those using blue dye to determine whether this method can be used to guild SLN biopsy and to assess its potential for SLN detection in early breast cancer. This is, to our best knowledge, the first demonstration of the use of carbon nanoparticles to detect SLNs in breast cancer.

## Methods

### Study Design

There were two steps in this research. Firstly, a pilot study was performed to determine the optimized condition using carbon nanoparticles for SLNs detection. Then, a prospective study was conducted to compare the sensitivity, specificity, and accuracy of using carbon nanoparticles for SLN mapping in breast cancer patients with those using blue dye.

### Ethics Statement

Patients with clinically node negative breast cancer were recruited to participate in this study, which was approved by Institutional Review Board of Fujian Provincial Tumor Hospital. Written informed consent was obtained prior to study participation.

### Patients

This study was designed to evaluate prospectively the feasibility of SLNs detection with carbon nanoparticles after peri-areolar intradermal injection of carbon nanoparticles. Inclusion criteria were: pathological diagnosis of breast cancer, maximum tumor diameter 3 cm, with indications for mastectomy or breast-conserving surgery, and no clinically positive axillary lymph nodes. Axillary lymph node status was assessed before surgery by ultrasonography. Exclusion criteria comprised palpable axillary lymph nodes, tumor diameter>3cm, multicentric tumor.

Demographic and clinicopathological data such as age, tumor size, grade, tumor histology, hormone receptor, HER2 status were recorded prospectively. The identification rate, sensitivity, specificity, accuracy, false negative rate, negative predictive value, positive predictive value was obtained after planned ALND.

### Carbon Nanoparticles Suspension

Carbon nanoparticles were purchased from Chongqing LUMMY Pharmaceutical Co (Chongqing, China) in the form of a standard carbon nanoparticles suspension (1ml: 50mg). This stable suspension of carbon pellets of 150 nm in diameter does not enter the blood circulation, causing no toxic side effects on the human body. A small amount of tiny carbon particles may be captured by macrophages, and are excreted through the lungs and intestines after a few months. Carbon nanoparticles do not cause acute systemic toxicity, either.

### Sentinel Lymph Node Biopsy

#### SLNs Identification Using Carbon Nanoparticles

SLNB was performed before breast conserving surgery or mastectomy as follows. First, a pilot study was performed to demonstrate the optimized condition using carbon nanoparticles for SLNs detection by investigating 36 clinically node negative breast cancer patients. 2 ml, 1 ml, 0.5 ml carbon nanoparticles suspension were used in 6 cases, respectively. The SLNs detection rate was evaluated with these doses. With the best dose, the best timing of injection was investigated in another 18 patients, who were divided into three groups according to the timing of injection (10–15 minutes before surgery, 1 day before surgery, 2 days before surgery). Each group contained 6 cases. The black-stained non-SLNs detection rate was assessed among different timing. Therefore, the best dose and timing of injection were determined. Second, in subsequent prospective study, 83 SLNB procedures were carried out using carbon nanoparticles suspension. The best dose of carbon nanoparticles suspension was intradermally injected into the periareolar region in 4 (clockwise) quadrants of the breast at the best injection time according to pilot study results. The whole breast was massaged for about 5 minutes to facilitate the absorption of carbon nanoparticles into the lymph vessels. In order to identify stained lymph nodes, a transverse incision was made just below the hair-bearing region of the axilla 10 minutes after dye injection. After raising the skin flaps, black stained lymphatic tracts were meticulously searched and traced towards axilla. The black stained lymph node to which a black stained lymphatic tract leads was considered as sentinel lymph node and excised along with perinodal fat. The specimen excised was sent for detailed pathological examination by paraffin fixation processing. All procedures of SLNB were finished within 30–45 minutes. Due to inherent cultural barriers and cancer fatalism in Chinese women, all patients chose complete axillary node dissection upon diagnosed with breast cancer. Therefore, a complete axillary lymph node clearance was done and the specimen was sent for histopathological examination. The information of full axillary clearance was used for validating the results of sentinel lymph node biopsy. The sentinel lymph nodes were formalin fixed and paraffin embedded. All SLNs were evaluated by Haematoxylin and Eosin (H&E) staining and those with negative histology for metastasis, immunohistochemical staining with monoclonal antibody against cytokeratin. Macrometastases were defined as a tumor size >2 mm and micrometastases as a tumor size between 0.2 and 2 mm. Tumors <0.2 mm were regarded as isolated tumor cell (ITC). A sentinel node was defined as positive if a macrometastases, micrometastases or ITC was identified.

#### SLNs Identification Using Blue Dye

Another 83 SLNB procedures were performed by using blue dye. Similarly, a 1% solution of blue dye (1 ml) was intradermally injected into the periareolar region in 4 (clockwise) quadrants of the breast. The whole breast was massaged for about 5 minutes. The blue stained lymph nodes were considered SLNs, which were harvested and then were sent for detailed pathological examination by paraffin fixation processing. A complete axillary lymph node clearance was done following SLNB and the specimen was sent for histopathological examination.

### Statistical Analysis

In pilot study, the detection rates of SLNs using different dose and the detection rates of non-SLNs in three injection timing were compared using the chi-squared test. In prospective study, the false negative rate (FNR), sensitivity, negative predictive value (NPV), positive predictive value (PPV), specificity, and accuracy of SLN biopsy with carbon nanoparticles and blue dye were calculated by using the following formulas:

False negative rate=number of false negative SLNs/(true positive+false negative nodes)x100

Sensitivity=number of true positive SLNs/(true positive+false negative nodes)x100

Negative predictive value=number of true negative SLNs/(true negative+false negative nodes)x100

Positive predictive value=number of true positive SLNs/(true positive+false positive nodes)x100

Specificity=number of true negative SLNs/(false positive+true negative nodes)x100

Accuracy=(true positive+true negative nodes)/total nodes x100

Variables of the methods were compared using the chi-squared test. IBM SPSS Statistics version 20 (SPSS Inc., Chicago, IL, USA) was used for statistical analyses. Values of P<0.05 were considered statistically significant.

## Results

### Injection Dosage and Timing of Application in Pilot Study

**Fig 1:**
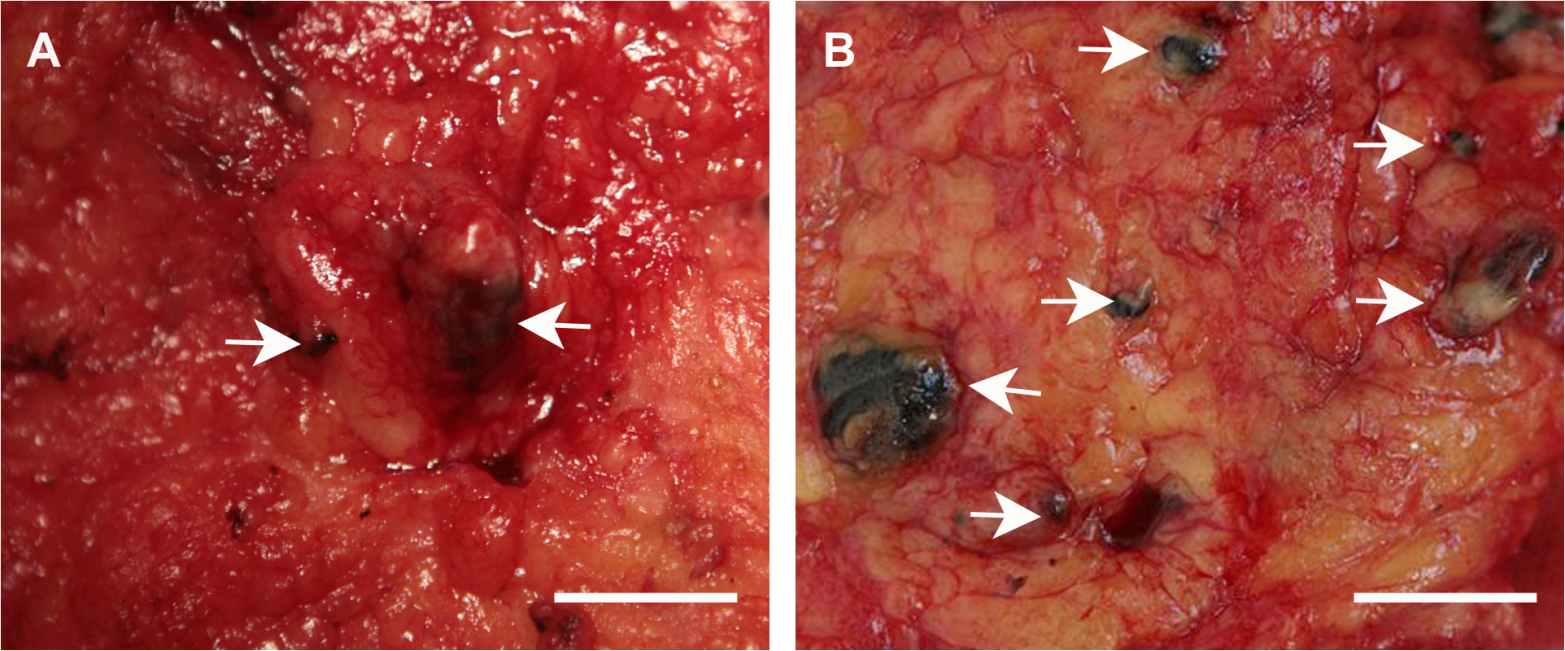
A Black-dyed lymph nodes (as indicated by arrows) were visible 1 day after injection of carbon nanoparticles. B Increased non SLNs (as indicated by arrows) were stained black by carbon nanoparticles when injected 2 days before surgery. Scale bar is 1cm.

In pilot study, 36 clinically node negative breast cancer patients were investigated. We used 2 ml carbon nanoparticles suspenstion at the beginning of study. We found non-SLNs had been dyed black with this dose, which affected correct SLN staging. Then we decreased the dose to 1 ml and 0.5 ml. The SLNs detection rate with 2 ml of carbon nanoparticles was 66.7% inferior to 100% using 1 ml of carbon nanoparticles. However, when the amount of carbon nanoparticles was decreased to 0.5 ml, the SLNs detection rate was reduced to 16.7% because carbon nanoparticles could not clearly show SLNs with this dose. There was statistically significant difference in the SLNs detection rate in these three doses (P = 0.01) (Table 1). When compared one by one, the SLNs identification rate was significantly higher in 1ml than in 0.5ml (p = 0.02). There was no difference in the identification rate between 1ml and 2ml (p = 0.46), nor was that between 2ml and 0.5ml (p = 0.24). Timing of application also affected the detection efficiency. With 1 ml of carbon nanoparticles, no black-stained non-SLNs were visible in patients who were injected 10–15 minutes before surgery (0/6) during complete axillary node dissection. In contrast, the black-stained non-SLNs detection rate was 83.3% (5/6) and 100% (6/6) in patients who were injected 1 day (Fig 1A) or 2 days (Fig 1B) before surgery, respectively. There was statistically significant difference in the non-SLNs detection rate in timing of application (P = 0.001) (Table 2). In terms of comparison of one by one, there was significant difference in non-SLNs detection rate between 10–15 minutes and 1day before surgery (p = 0.02), so was that between 10–15 minutes and 2day before surgery (p = 0.002). No statistically significant differences in non-SLNs detection rates were seen between 1day and 2day before surgery (p = 1.0).

**Table 1:** Comparison of SLNs detection rates using carbon nanoparticles between different doses in pilot study.

Injected dose	SLN detection rate	P value (Chi-square)
2ml	66.7%(4/6)	0.01
1 ml	100%(6/6)	
**0.5 ml**	**16.7%(1/6)**	

**Table 2:** Comparison of non-SLNs detection rates using carbon nanoparticles between different injection timing in pilot study.

Timing of injection	Non-SLN detection rate	P value (Chi-square)
10–15min before surgery	0%(0/6)	0.001
1day before surgery	83.3%(5/6)	
2day before surgery	100%(6/6)	

### Patients and Tumor Characteristics in Prospective Study

Eighty three women with operable primary breast cancer took part in carbon nanoparticles groups. Their median age was 51 years (range 28–75 years). Of these 83 patients, 46 were premenopausal and 37 were postmenopausal. Among these 83 patients, 49 had tumor less than 2 cm, 32 had tumor larger than 2 cm and 2 were Tx tumors (previous surgical excision at outside our institution). There were another 83 cases of operable primary breast cancers using blue dye technique. Their median age was 49.2 years (range 24–72 years). Of these 83 patients, 43 were premenopausal and 40 were postmenopausal. Patient data in prospective study are shown in Table 3.

**Table 3:** Patients and tumor characteristics in prospective study.

Characteristics	Carbon nanoparticles groups (n = 83)	Blue dye groups (n = 83)
Age		
<50	46(55.4%)	43(51.8%)
>50	37(44.6%)	40(48.2%)
Tumor size		
<2cm	49(59%)	45(54.2%)
>2cm	32(38.6%)	37(44.6%)
Tx	2(2.4%)	1(1.2%)
Grade		
I	34(41%)	36(43.4%)
II	26(31.3%)	29(34.9%)
III	23(27.7%)	18(21.7%)
Tumor histology		
IDC	67(80.7%)	62(74.7%)
ILC	16(19.3%)	21(25.3%)
Estrogen receptor status		
Positive	58(69.9%)	59(71.1%)
Negative	25(30.1%)	24(28.9%)
HER2 status		
Positive	20(24.1%)	22(26.5%)
Negative	63(75.9%)	61(73.5%)

IDC: Invasive ductal carcinoma; ILC: Invasive lobular carcinoma; HER2: Human epithelial growth factor receptor2.

### Sentinel Lymph Node Biopsy in Prospective Study

**Fig 2:**
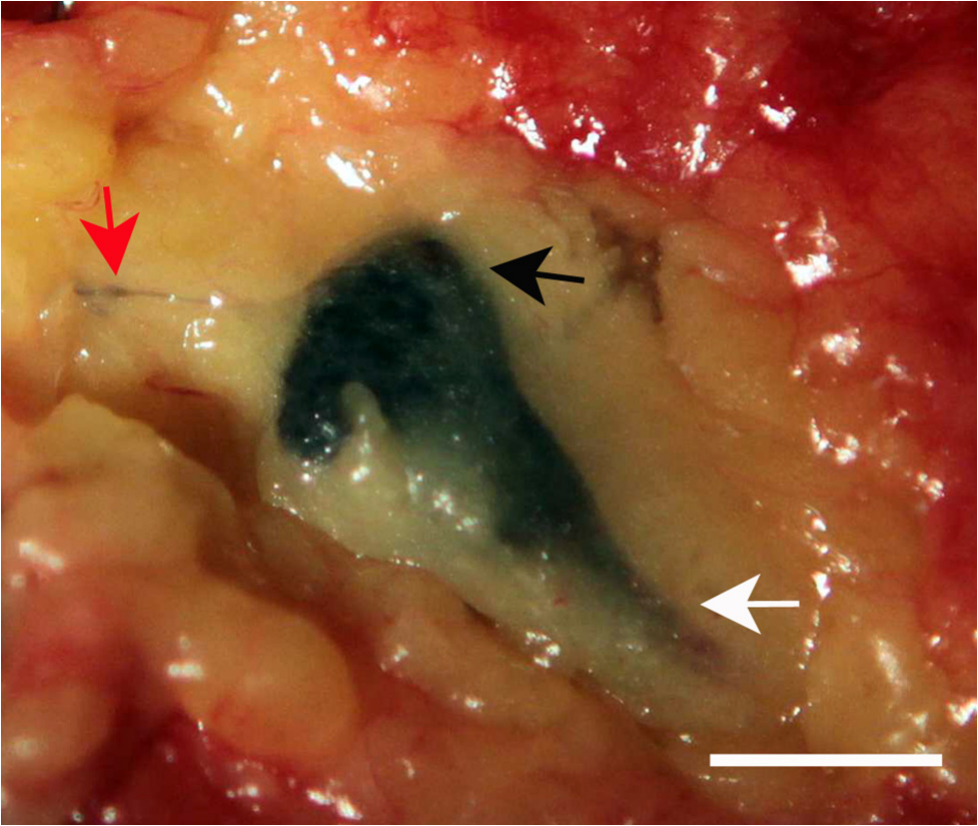
A black-stained SLN (as indicated by black arrow) with afferent lymph vessel (as indicated by white arrow) and efferent lymph vessel (as indicated by red arrow). Scale bar is 1cm.

The carbon nanoparticles technique was found to be as easy to use as blue dye method by the surgeons. This method did not require special equipment in the operating room to enable this procedure. In prospective study, SLNs were successfully identified in all patients (100%) using carbon nanoparticles method. All SLNs had been stained black by carbon nanoparticles (Fig 2). The mean number of sentinel nodes per patient was 2.9 (range, 1–10). None of the 83 patients experienced adverse effects in response to carbon nanoparticles. Of the 83 SLNB procedures using blue dye, 73 had SLNs successfully identified, an identification rate of 88% (73/83). The average number of SLNs detected using blue dye was 2.0 (range, 1–6). We observed no allergic reactions to blue dye during the study.

### Histopathology in Prospective Study

**Fig 3:**
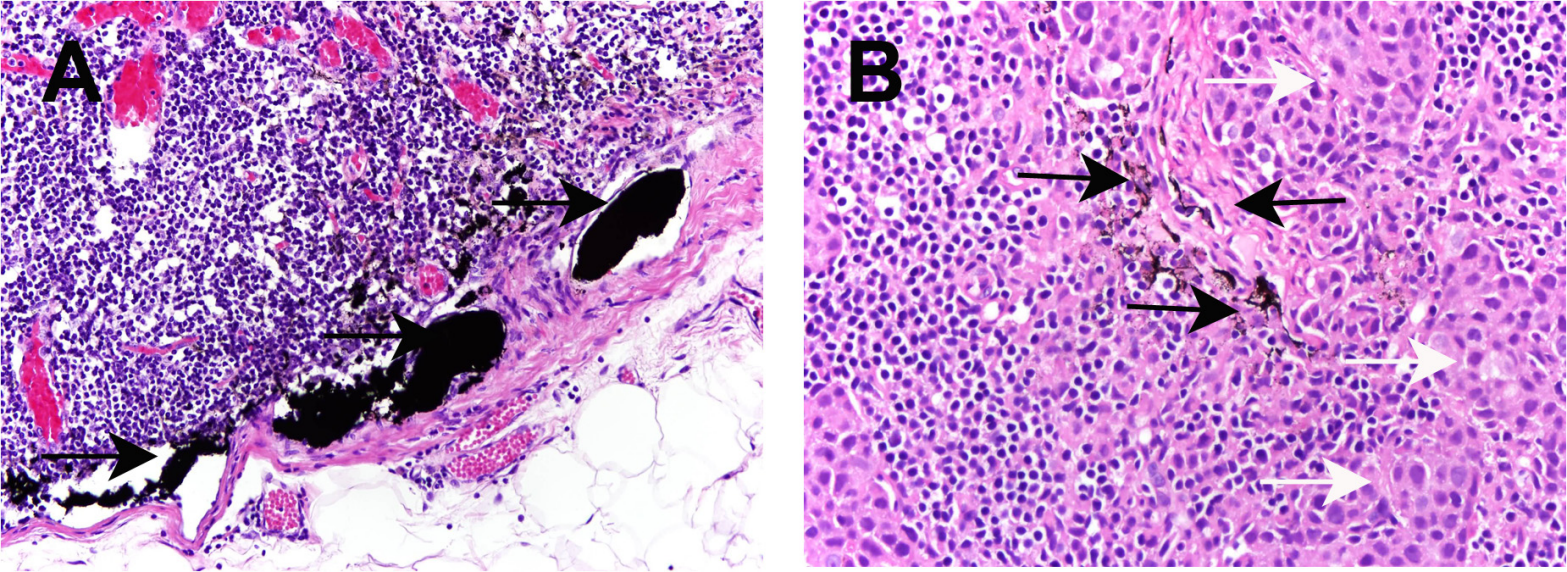
Carbon nanoparticles in SLNs in H-E staining images. A Carbon nanoparticles (as indicated by black arrow) in afferent lymphatic vessel and lymphoid sinus in negative SLNs (x20); B Carbon nanoparticles (as indicated by black arrow) in positive SLNs. White arrow indicated cancer cells (x20).

Carbon nanoparticles were seen in lymphatic vessels, and lymphoid sinus in negative SLNs. Similarly, carbon nanoparticles were seen in positive lymph nodes (Fig 3). Of the 83 patients with sentinel nodes using carbon nanoparticles, 24 (28.9%) had a tumor-positive SNLB specimen. Among these 24 patients, 19 (79.2%) had at least 1 macrometastasis, and 3 (12.5%) had at least 1 micrometastasis as the largest metastatic deposit. Another 2 (8.3%) had individual tumor cells. Immunohistochemistry was performed in patients with negative histology for metastasis. Of the 59 patients who had negative sentinel nodes, all axillary nodes were negative in 56 cases. The remaining three patients with disease-free sentinel nodes were tumor-positive in other axillary nodes. Thus, with carbon nanoparticles technique, the negative predictive value was 94.9% (56/59), the sensitivity = 88.9% (24/27), false negative rate = 11.1% (3/27), specificity = 100% (56/56), the positive predictive value = 100% (24/24), and accuracy being 96.4% (80/83) (Table 4). Of the 83 SLNB procedures using blue dye, 73 had SLNs successfully identified, an identification rate of 88% (73/83). Among these 73 patients, 16 (21.9%) had a tumor-positive SNLB specimen including 12 with at least 1 macrometastasis, 3 with at least 1 micrometastasis. Another one had individual tumor cells. 3 patients with positive non-SLNs had no SLNs involvement according to the final histology; Thus, the false negative rate was15.8% (3/19), the sensitivity = 84.2% (16/19), specificity = 100% (54/54), the positive predictive value = 100% (16/16), and accuracy being 95.9% (70/73) (Table 5). The SLNs identification rate using carbon nanoparticles was significantly higher than that using blue dye (p = 0.001). In terms of accuracy and false negative rate, there were no statistically significant differences in between the two methods (Table 6). However, there was a decrease in the false negative rate, from 15.8% with blue dye to 11.1% using carbon nanoparticles.

**Table 4:** Accuracy of SLNB using carbon nanoparticles in early breast cancer.

		ALND		Total	
		Positive	Negative		
SLNB status	Positive	24	0	24	PPV = 100%(24/24)
	Negative	3	56	59	NPV = 94.9%(56/59)
	Total	27	56	83	
		Sens = 88.9% (24/27)	Spec = 100% (56/56)	Accuracy = 96.4%(80/83)	

Sens: Sensitivity; Spec: Specificity; PPV: Positive predictive value; NPV: Negative predictive value.

**Table 5:** Accuracy of SLNB using blue dye in early breast cancer.

		ALND		Total	
		Positive	Negative		
SLNB status	Positive	16	0	16	PPV = 100%(16/16)
	Negative	3	54	57	NPV = 94.7%(54/57)
	Total	19	54	73	
		Sens = 84.2%(16/19)	Spec = 100% (54/54)	Accuracy = 95.9%(70/73)	

Sens: Sensitivity; Spec: Specificity; PPV: Positive predictive value; NPV: Negative predictive value.

**Table 6:** Comparison of SLN detection rates between using carbon nanoparticles and blue dye in prospective study.

	carbon nanoparticles	Blue dye	P value
SLN identification rate	100%	88%	0.001
Accuracy	96.4%	95.9%	1.0
False negative rate	11.1%	15.8%	0.68

## Discussion

Since the first introduction in the 1990s, sentinel lymph node biopsy has been the standard of care for breast cancer patients with clinically negative axilla, which is less invasive, less morbid but with an efficacy equal to ALND [2–4]. Current existing techniques, including blue dye, radioisotope, combined technique, and ICG, are useful in identification of SLNs. However, the optimal modality remains challenging because of certain disadvantages with these techniques.

The present study confirmed that carbon nanoparticles can be used successfully for SLNs identification in patients with early breast cancer. Carbon nanoparticles have a strong affinity for the lymphatic system. Once injected into the subareolar tissue, they were taken up specifically by lymphatic vessels and delivered to the sentinel lymph nodes. The lymph nodes then turned black (Fig 2), which facilitated their identification during surgery. Carbon nanoparticles are a popular nanomaterial and readily available. It rarely produces side effects and we observed no allergic reactions during the study. The identification rate with carbon nanoparticles technique was better than using blue dye in our study, although the accuracy of detection between both techniques was comparable (p = 1.0). In addition, carbon nanoparticles were superior to a combination of gamma probe and blue dye in SLNs identification rate. The latter was currently reported to obtain the best SLNs detection rate [20]. The false negative rate is another common index of sentinel node mapping success. Although the false negative rates were not statistically different between the two methods in this study (Table 5), the reduced false negative rate with the use of carbon nanoparticles may provide favorable clinical benefit. With increasing experience of SLNs detection using carbon nanoparticles, this method may hold great promise for SLNs detection due to improved identification rates and lower false-negative rates.

An optimal lymphatic tracer should have size (in the range of 50–200 nm) small enough to enter the lymphatic capillaries and transport rapidly to the SLNs, yet large enough to retain in the sentinel nodes long enough for imaging and SLNs identification without prematurely migrating to higher tier nodes [21–23]. Nanosized carbon particles with an average diameter of 150 nm, which ensures that these particles pass through the lymphatic capillaries and accumulate in the lymph nodes long enough for the SLNs to be identified during surgery. In contrast, the blue dye molecules are rather small (<2 nm), and thus they can quickly transport through the sentinel lymph nodes, causing color fading of blue dye and a high possibility of false negative rate [24], as is the case with ICG [15]. Therefore, it should be more easy applying carbon nanoparticles than using blue dye or ICG in SLN biopsy due to its longer presentation time in SLNs. This has important clinical implications. Because the dyes quickly diffuse through SLNs, a ‘blue’ node may not be the true sentinel node, but instead a level II or even level III, non-sentinel node. Therefore, non-sentinel lymph nodes could incorrectly be identified as SLNs, causing more nodes than necessary to be excised and a false-negative staging. Currently, approximately 1% of the nodes are undetectable when using a radiotracer and a blue dye during sentinel lymph node biopsy [25], probably because radiotracer or blue dye flows through the SLNs to higher tier nodes. A better retention in the SLNs using carbon nanoparticles is most likely to reduce this false negative detection. In this case, carbon nanoparticles detection was more reliable and stable than blue dye or ICG because the dye distribution in SLNs subsequent to injection of carbon nanoparticles was more likely to last longer.

The timing of the application differs with different tracer. With the patent blue dye, the maximum coloring is obtained the tenth minute after injection. After that, the coloring gradually fades, indicating SLNB procedure using blue dye should be finished within ten minutes. In our pilot study, we had injected the carbon nanoparticles at 10–15 minutes, 1 day, 2 days before surgery. We found that 10–15 minutes before surgery is the best time for maximum coloring of SLNs, and black SLNs could be identified very clearly during the surgery (Table 2). Following SLNB which was finished within 20–30 minutes, no black-stained non-SLNs were visible during complete axillary node dissection. However, the time lapse of carbon nanoparticles retention in SLNs needs to be further investigated. Notably, increased non sentinel lymph nodes were stained black 1 day or 2 days after injection of carbon nanoparticles (Fig 1). In contrast, data from Yuan et al.'s study [26] demonstrated all the patent blue-dyed nodes lost the color rapidly when the timing of injection was more than 6 h before surgery. Another critical issue in SLNs identification is injection dose. In our pilot study, we used 2 ml carbon nanoparticles suspenstion at the beginning of study. We found non-SLNs had been dyed black with this dose and then we decreased the dose gradually. On the contrary, 0.5 milliliter carbon nanoparticles sometimes could not clearly show SLNs, which affected detection efficiency. Therefore, a 1 ml carbon nanoparticles suspension was sufficient and recommended for identification of SLNs (Table 1).

Of the 59 patients with a negative SLN using carbon nanoparticles suspension, metastasis was found in a non-sentinel node in three patients. We reviewed the characteristics of these patients. One had a 30-mm grade 3 invasive ductal carcinoma which has more likely to skip the SLNs and metastasize to high-level station lymph nodes. One tumor was located medially and preferentially drained to the internal mammary nodes (IMNs) instead of the axillary nodes. In this study, lymph nodes existing outside the axilla were not examined. The other patient had undergone previous surgery which could have disrupted lymphatic drainage to the axillary nodes. Therefore, tumor size, localization, previous surgery could have negative effects on the accuracy of the SLN biopsy [27–29].

This study used gold standard- pathological analysis, to distinguish the positive and negative SNLs with an aim at investigation of the feasibility of using carbon nanoparticles to detect SLNs in early breast cancer. Our results showed that carbon nanoparticles could be an excellent candidate to stably and reliably detect SLNs in patients with early breast cancer.

The detection rate using carbon nanoparticles was 100%, with lower false negative rate than blue dye. This demonstrated that SLNs detection with our system was practical and applicable, especially in country where the incidence of newly diagnosed breast cancer is rising and the use of the sentinel lymph node biopsy procedure is limited due to less access to radioisotopes.

The use of carbon nanoparticles does have some limitations. For instance, carbon nanoparticles cannot be seen through skin and fatty tissue, and permit only limited visualization of afferent lymphatic vessels and the SLNs. However, fluorescent carbon nanoparticles obtained by conjugating carbon nanoparticles and ICG, holds great promise for SLNs mapping, which allows accurate localization of SLNs. Another disadvantage of carbon nanoparticles is tattooing of the breast which was observed in 10 cases, with complete resolution within 2 months in each case.

In summary, this prospective study demonstrated feasibility and accuracy of using carbon nanoparticles for SLN mapping in breast cancer patients. The carbon nanoparticles method would be particularly useful in institutions without access to radioisotope.
